# Long-term effects on nitrogen and benthic fauna of extreme weather events: Examples from two Swedish headwater streams

**DOI:** 10.1007/s13280-014-0562-3

**Published:** 2014-11-15

**Authors:** Stefan Löfgren, Ulf Grandin, Sonja Stendera

**Affiliations:** 1 Department of Aquatic Sciences and Assessment, Swedish University of Agricultural Sciences, SLU, P.O. Box 7050, 750 07 Uppsala, Sweden; 2 Weinsbergtalstraße 81, 42657 Solingen, Germany

**Keywords:** Storm-felling, Bark beetle, Nitrate, Rainstorm, Flashflood, Benthic macroinvertebrates

## Abstract

Climate change is expected to cause an increased frequency of extreme events such as heavy floods and major storms. Such stochastic events have an immediate impact on surface water quality, but the long-term effects are largely unknown. In this study, we assess long-term monitoring data from two Swedish headwater catchments affected by extreme weather events. At one site, where nitrogen effects in soil water, groundwater, and stream water were studied after storm-felling and subsequent forest dieback from bark beetle attack, long-term (>5 years) but relatively modest (generally <1 mg L^−1^) increases in ammonium (NH_4_-N) and nitrate (NO_3_-N) concentrations were observed in the various aqueous media. At the other site, where effects on benthic fauna were studied in a stream impacted by extreme geophysical disturbances caused by rainstorm-induced flashflood, only short-term (1 year) effects were revealed both regarding diversity and composition of species.

**Electronic supplementary material:**

The online version of this article (doi:10.1007/s13280-014-0562-3) contains supplementary material, which is available to authorized users.

## Introduction

Climate change is predicted to increase air temperature by 2–5 °C and precipitation by 9–17 % in the Baltic region by the end of twenty-first century (BACC 2008). Increased frequencies of extreme events such as heavy floods, long droughts, or major hurricanes are also expected according to IPCC (2012). Such stochastic events may have an immediate effect on the boreal landscape as well as long-lasting cascading impacts coupled with succeeding insect- and fungi-induced tree mortality, affecting biogeophysical and biogeochemical processes both in terrestrial and aquatic environments (Edburg et al. 2012; Mikkelson et al. 2013).

Natural disturbances such as storm felling and bark beetle outbreaks have similarities with tree harvesting. A diminished forest canopy reduces water and nutrient uptakes by the vegetation, creating prerequisites for increased element leakage to streams. However, a typical bark beetle outbreak after massive storm felling lasts for about 3–5 years and mainly affects mature trees (Schroeder and Lindelöw 2002; Grodzki et al. 2006; Långström et al. 2009), while final felling is more or less instantaneous and also includes cutting of young trees. The prolonged and initially scattered canopy removal in bark beetle-infested stands results in less stark changes in the biogeochemistry compared with final felling due to compensatory capacity associated with undisturbed residual vegetation and soils (Tahovská et al. 2010; Griffin et al. 2013; Kaňa et al. 2013; Rhoades et al. 2013). However, bark beetle-induced forest dieback may tangibly affect the surface water concentrations of nitrogen (N), phosphorus (P), dissolved organic carbon (DOC), and acidity-related elements such as base cations and aluminum (Mikkelson et al. 2013; Oulehle et al. 2013; Vrba et al. 2014).

Following bark beetle attacks, N-limited catchments in North America generally showed small effects on the nitrate (NO_3_^−^) response in both soil and surface waters, while N-rich catchments in Central Europe and Asia exhibited much larger responses (Huber 2005; Mikkelson et al. 2013). Ammonium (NH_4_^+^) concentrations in soil water (SW) showed a more profound, universal increasing trend. Depending on residual vegetation, regrowth, N turnover, hydrologic flow paths, etc., it is possible that the N concentrations in surface waters will remain unaffected by bark beetle outbreaks (Mikkelson et al. 2013).

Besides biogeochemical effects, natural disturbances play a major role in structuring most ecological communities by redistribution of available resources. In lotic systems, hydrologic events such as floods accompanied by streambed and thus substratum movement are pervasive in forming benthic assemblages (e.g., Resh et al. 1988; Poff 1992; Townsend et al. 1997). Hydrologic disturbances can have different impacts on stream biota, depending on their intensity and frequency as well as dynamics (Lake 2000, 2003), timing and duration (Peterson and Stevenson 1992) and are influenced by e.g., structural heterogeneity (e.g., Hart and Finelli 1999; Boix et al. 2010), and channel morphology (Lake 2007). Hydrologic disturbances therefore act as environmental filters forcing a high selection pressure on stream biota (Poff 1997), and species must have certain adaptations supporting their resistance and resilience to persist in frequently disturbed streams. Species possessing these adaptations are likely to dominate communities (Death and Winterbourn 1994), especially in frequently disturbed, unstable, albeit predictable environments rapidly colonizing post-event habitats and recovering to pre-event densities (Fritz and Dodds 2004).

**Fig. 1:**
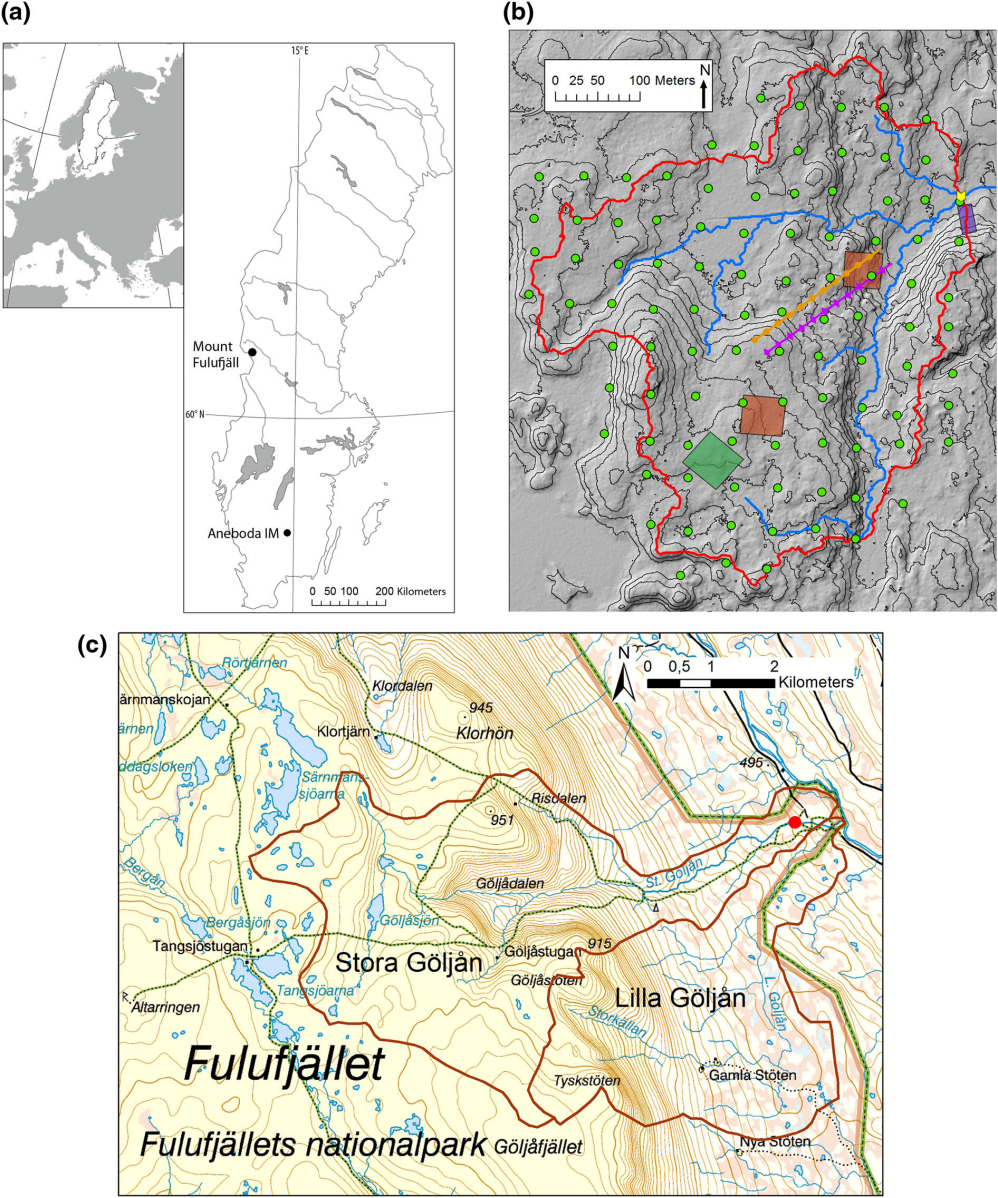
**a** Map showing the locations of Mount Fulufjäll (Stora Göljån stream) and Aneboda IM. **b** Generalized map of the Aneboda catchment showing the approximate location of the sampling stations. *Red line* catchment water divide; *blue line* stream; *orange area* or *line with circles* litterfall; *purple area* or *line with diamonds* throughfall; *dark green area* soil sampling plot; *brown area* vegetation plot; *blue squares* or *line with squares* GW sampling; *pink cross* or *line with cross* soil water sampling; *green rings* circular plots for monitoring tree layer, needle chemistry, algae, and lichen cover on needles; *yellow arrow* hydrology and water chemistry in catchment outlet. Contours at 2-m intervals. **c** Generalized map of the Stora Göljån and Lilla Göljån catchments showing the approximate location of the stream water and benthic invertebrate sampling stations. *Red line* catchment water divide; *blue line* stream. Contours at 20-m intervals. Background map: © Lantmäteriet, i2012/901

In this study, we have assessed the impacts of stochastic weather events by using long-term monitoring data from two Swedish headwater streams (Fig. 1a) affected by a hurricane and a rainstorm, respectively. Both events occurred at sites with seminatural headwater catchments in protected areas with unmanaged forests.

**Fig. 2:**
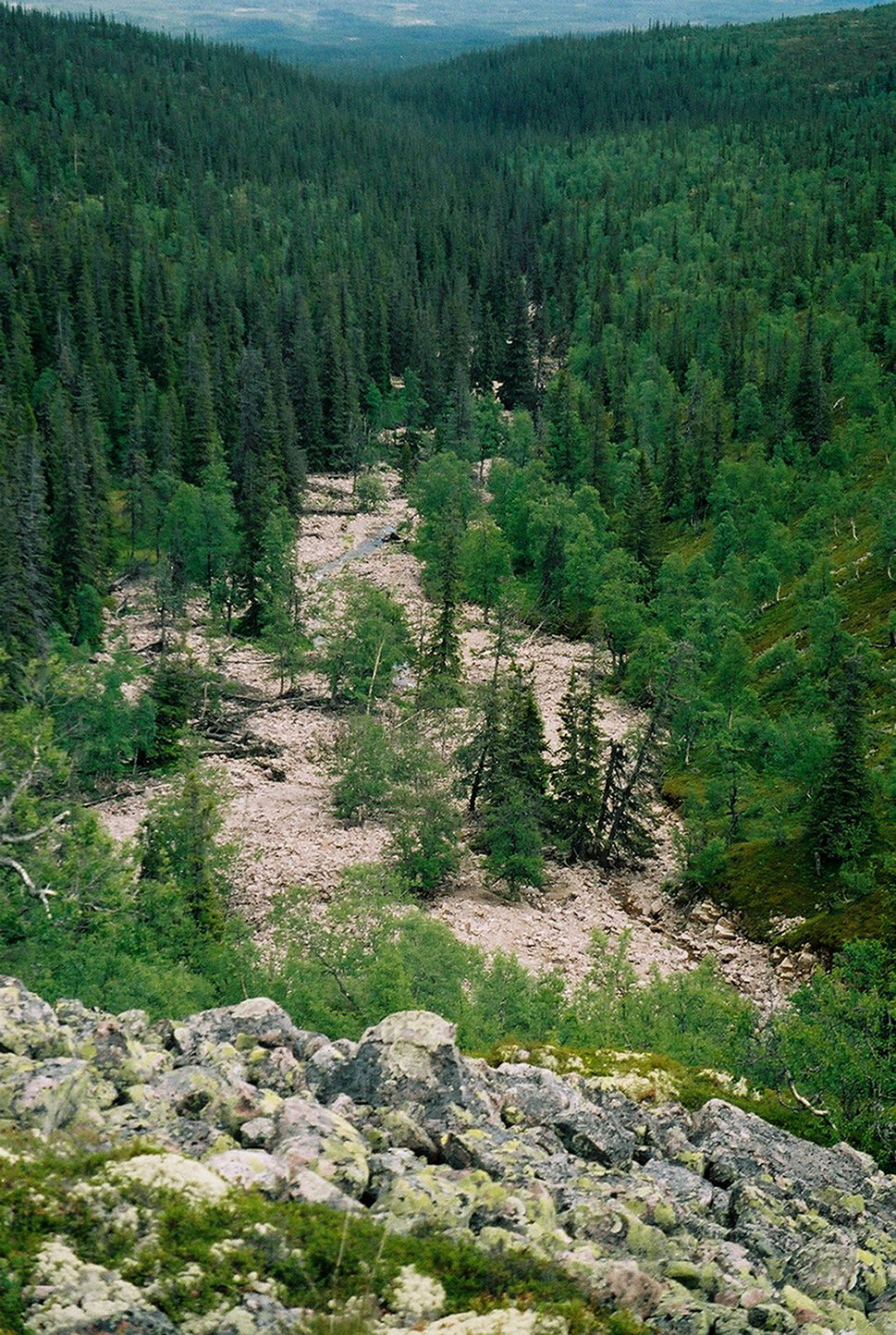
The Stora Göljån stream valley downstream the mountain plateau in June 2004. The exposed mineral surfaces were created by erosion and sedimentation during the flashflood. Photo: Stefan Löfgren, SLU

**Fig. 3:**
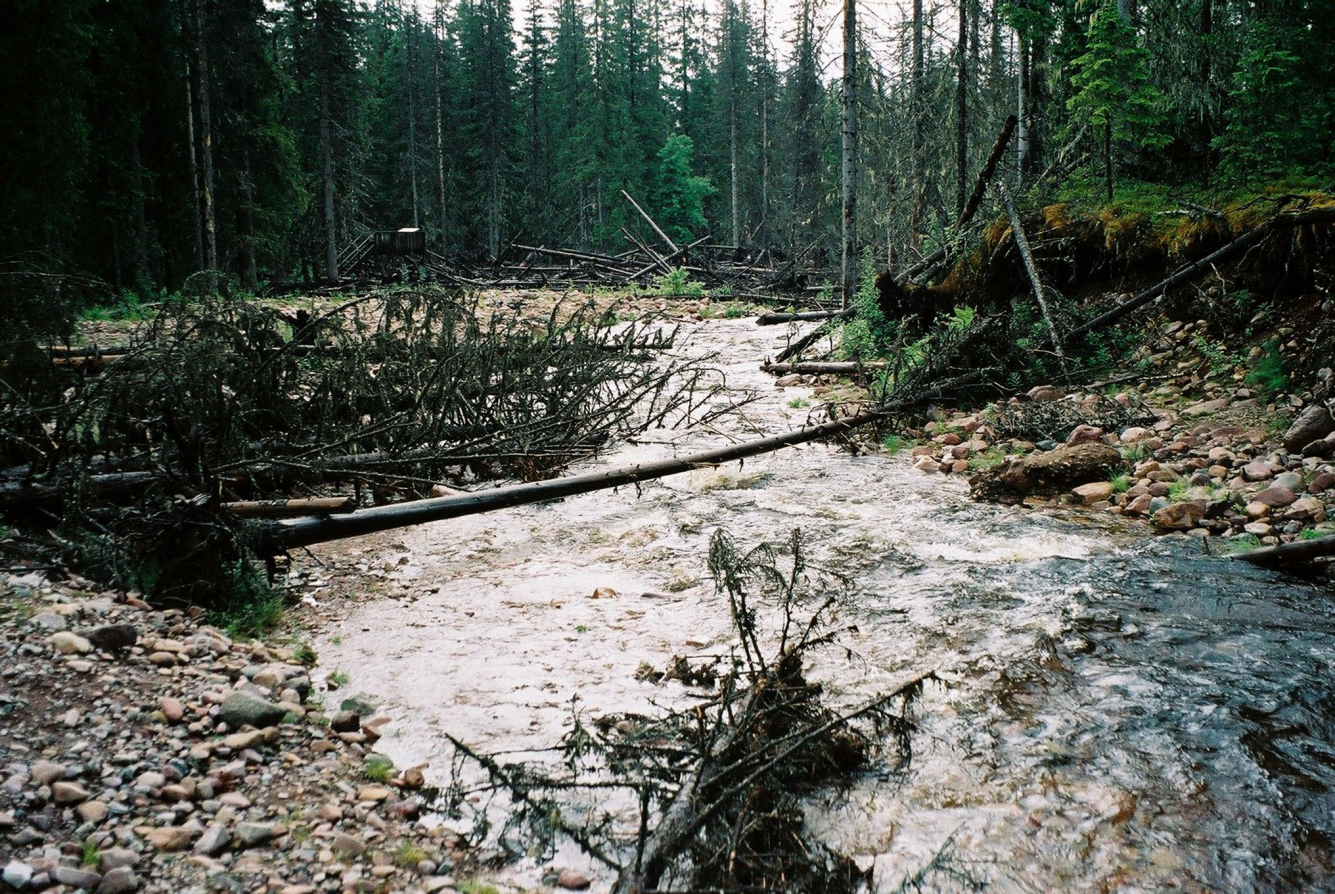
Flashflood-created new stream channel just downstream of the benthic fauna sampling site in Stora Göljån in June 2004. Photo: Stefan Löfgren, SLU

In January 2005, the integrated monitoring (IM) site at Aneboda was hit by the storm Gudrun. Most of the mature Norway spruce trees (*Picea abies* (L.) Karst.) were either storm felled or killed by the succeeding bark beetle (*Ips typographus* L.) outbreak. In August 1997, a rainstorm hit Mount Fulufjäll, and the resulting flashflood in the stream Stora Göljån completely changed the streambed geomorphology and uprooted trees and other vegetation along broad riparian strips (Figs. 2, 3). This was followed by attacks of bark- and wood-boring beetles, killing standing trees along the riparian forest edge. At both Aneboda and Stora Göljån, salvage felling did not occur, and the dead vegetation was left at site.

We have used monitoring data to investigate the impacts of (i) storm felling and extensive bark beetle-induced forest dieback on the N concentrations in SW, groundwater (GW), and stream water at Aneboda IM and; and (ii) a flashflood, and subsequently strong geomorphologic disturbance on benthic macroinvertebrates and their recovery patterns in Stora Göljån by tracking the temporal changes in assemblage composition structure and certain metrics (i.e., diversity, abundance, taxa richness) during the years before and after the event at Mount Fulufjäll. We predicted (i) strong and long-term effects on benthic macroinvertebrates in Stora Göljån due to the extreme geophysical disturbance, and (ii) no effects on the N concentrations in the Aneboda IM stream with low pre-event N-concentrations.

## Study areas and weather events

### Aneboda IM

Aneboda IM is a small (18.9 ha) forested catchment located above the highest marine coastline (210–240 m a.s.l.) in south central Sweden (Fig. 1b) and one of four Swedish IM sites. In Starr (2011), detailed descriptions of the catchments, methods, and results from the IM sites are presented. At Aneboda IM, the average atmospheric deposition of N on open field is around 7.5 kg ha^−1^ year^−1^, but as only one third of that averagely is present in throughfall (Table 1), it indicates a N-limited forest ecosystem. Before the catastrophic storm in 2005, nonmanaged, multi-aged Norway spruce covered 73 % of the catchment.

**Table 1:** Catchment characteristics for Stora Göljån and Aneboda IM

	Stora Göljån^a^	Aneboda IM^b^
Catchment area (ha)	2180	18.9
Latitude	N61°34′	N57°05′
Longitude	E12°54′	E14°32′
Altitude (m a.s.l.)	550–980	210–240
Bedrock geology	Sandstone	Granite
Quaternary deposit	Glacial till	Glacial till
Mean annual temperature (°C)	1.4	5.8
Precipitation (mm year^−1^)	835	750
Snow cover (days)	175–200	110
Tree limit (m a.s.l.)	850	–
Deposition, mean 1995–2012^c^		
Open field (kg S ha^−1^ year^−1^)	1.8	3.7
Throughfall (kg S ha^−1^ year^−1^)	1.4	3.6
Open field (kg N ha^−1^ year^−1^)	1.8	7.5
Throughfall (kg N ha^−1^ year^−1^)	0.8	2.4
Land cover (% of catchment area)		
Alpine heath	65	0
Deciduous trees	6	24
Norway spruce	20	73
Scots pine	5	3
Wetland	2	0
Lake area	2	0

^a^Data from Snäll (1997)

^b^Data from Löfgren et al. (2011)

^c^Data from the Swedish Throughfall Network (krondroppsnatet.ivl.se) and Swedish IM (unpubl. data)

During January 8–9, 2005, southern Sweden was hit by a major storm named Gudrun. Wind speeds peaking at 42 m s^−1^ were registered on the Swedish west coast (Anonymous 2006). At the inland, a peak wind speed of 33 m s^−1^ was recorded at the meteorological station at the city of Växjö, located 30 km south of Aneboda IM, where maximum wind speeds >20 m s^−1^ were recorded over 9 h (unpublished data). Denmark, the Baltic states, and Russia were also affected, but to a much lower extent (Haanpää et al. 2006).

**Fig. 4:**
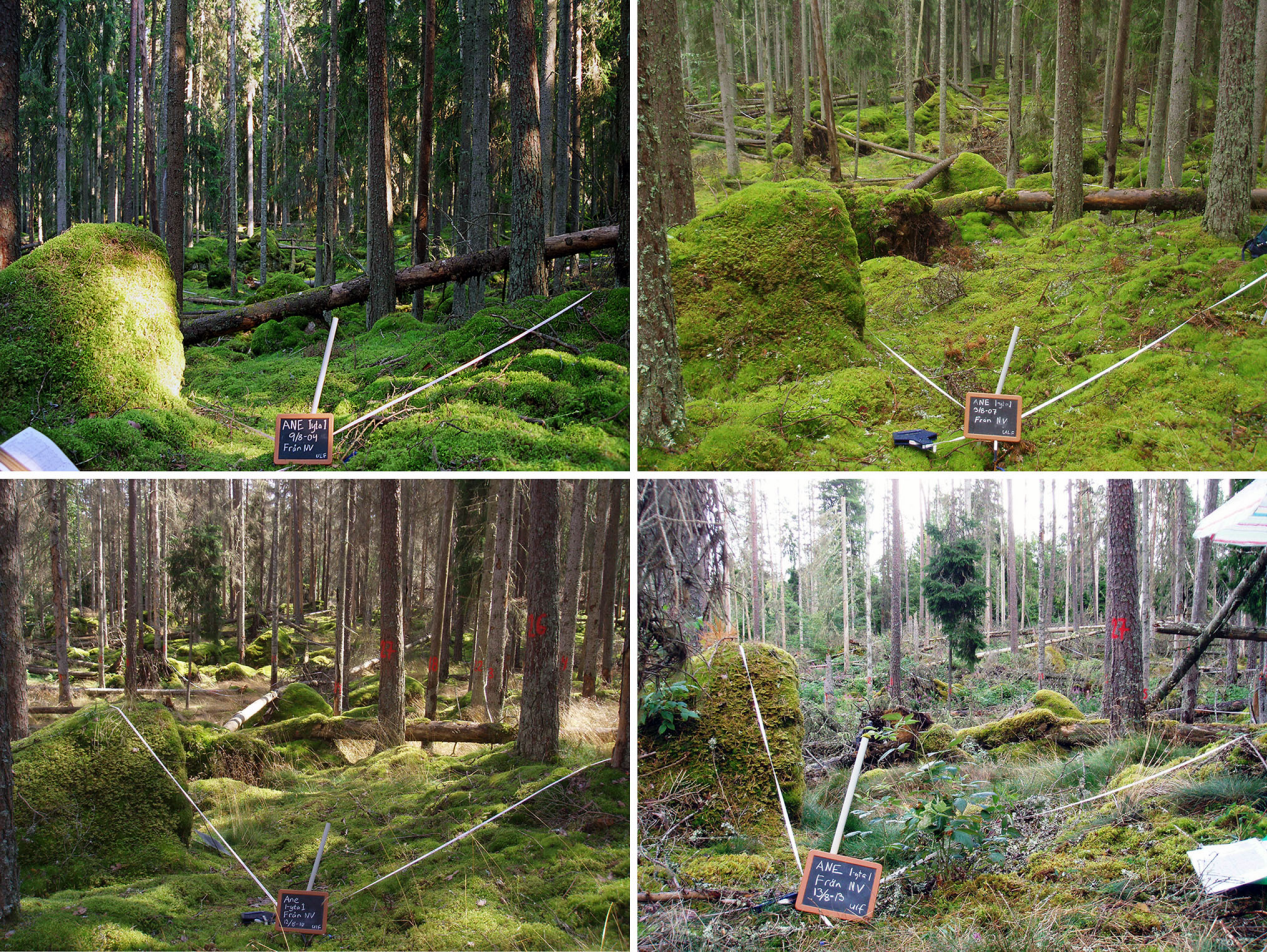
The intensive vegetation plot no 1 at Aneboda IM viewed from the northwest corner in the middle of August in 2004 (*upper left*), 2007 (*upper right*), 2010 (*lower left*), and 2013 (*lower right*). The major storm Gudrun hit the area in January 2005, and the bark beetle infestation became visible in 2008. Photo: Ulf Grandin, SLU

In Sweden, approximately 75 million m^3^ (stem volume over bark) of trees were felled during this night, corresponding to twice the normal annual cut in the affected area (Valinger and Fridman 2011). About 272 000 ha of forest was severely affected by the storm. The most devastating storm felling occurred in south-central Sweden, where a volume of 65–75 m^3^ ha^−1^ was damaged (Anonymous 2006). The highest impact was on mature even-aged Norway spruce stands (Anonymous 2006). In the Aneboda IM catchment, about 15–20 % of the trees were felled by the storm. However, the downed Norway spruce attracted bark beetles (*Ips typographus* L.), which caused a massive outbreak where the beetles infested a major part of the Norway spruce trees that survived the storm (Fig. 4). The quantitative effects of the storm felling and bark beetle infestation on the tree layer are assessed below.

### Stora Göljån

Mount Fulufjäll, national park since 2002, is located on the border to Norway in the southern part of the Swedish alpine region with a maximum altitude of 1042 m a.s.l. (Fig. 1c). At elevations above 900 m a.s.l., the mountain resembles a plateau covered by dry heaths with lichens and grass heaths. Fairly steep slopes descend to the surrounding land from about 400 to 500 m a.s.l. Mountain birch species (*Betula pubescens* ssp. *czerepanovii* (N. I. Orlova) Hämet-Ahti) reach from 800 to 925 m a.s.l., whereas Norway spruce dominates the lower parts of the slopes. The headwater stream Stora Göljån begins on a plateau at ≈1000 m a.s.l. and discharges on the eastern side of Mount Fulufjäll in River Fulan. The catchment area is 21.8 km^2^ with alpine heath as the dominant land cover (Table 1). An old (>160 years) nonmanaged Norway spruce forest covers the lower parts of the catchment (31 %, Fig. 2) (Snäll 1997).

During August 30–31, 1997, a rainstorm hit Mount Fulufjäll, and a new Swedish precipitation record of 276 mm within 24 h was measured at Lake Rösjön on the plateau. Further south on the eastern side of the mountain, an even higher precipitation of 300–400 mm 24 h^−1^ was estimated (Vedin et al. 1999). The resulting flashflood increased the total water discharge in Stora Göljån and the neighboring stream Lilla Göljån (total catchment area 35.9 km^2^) from an annual average discharge of 0.4 to about 300 m^3^ s^−1^ at the joint outlet in Fulan (Vedin et al. 1999). The amount is comparable with the annual average discharge of 353 m^3^ s^−1^ in the River Dalälven outlet at Bothnian Sea (catchment area 28 919 km^2^). The flashflood completely changed the streambed geomorphology downstream the plateau and created new flood channels in the coarse alluvium (Borgström et al. 1999). The erosive force was enormous, and the flood transported large boulders (>1 m^3^) over distances up to 100 m downstream and cleared the vegetation along broad riparian strips (often up to 50 m broad clearings). Damming caused by piles of uprooted trees and other vegetation trapped course and fine sediments, which at some occasions during the flood raised the water level up to 8.5 m from the present level and expanded the stream width in the order of 200–250 m (Borgström et al. 1999). For a detailed description of the meteorological, hydrologic, and geomorphologic effects of this event, see Vedin et al. (1999) and Borgström et al. (1999).

**Fig. 5:**
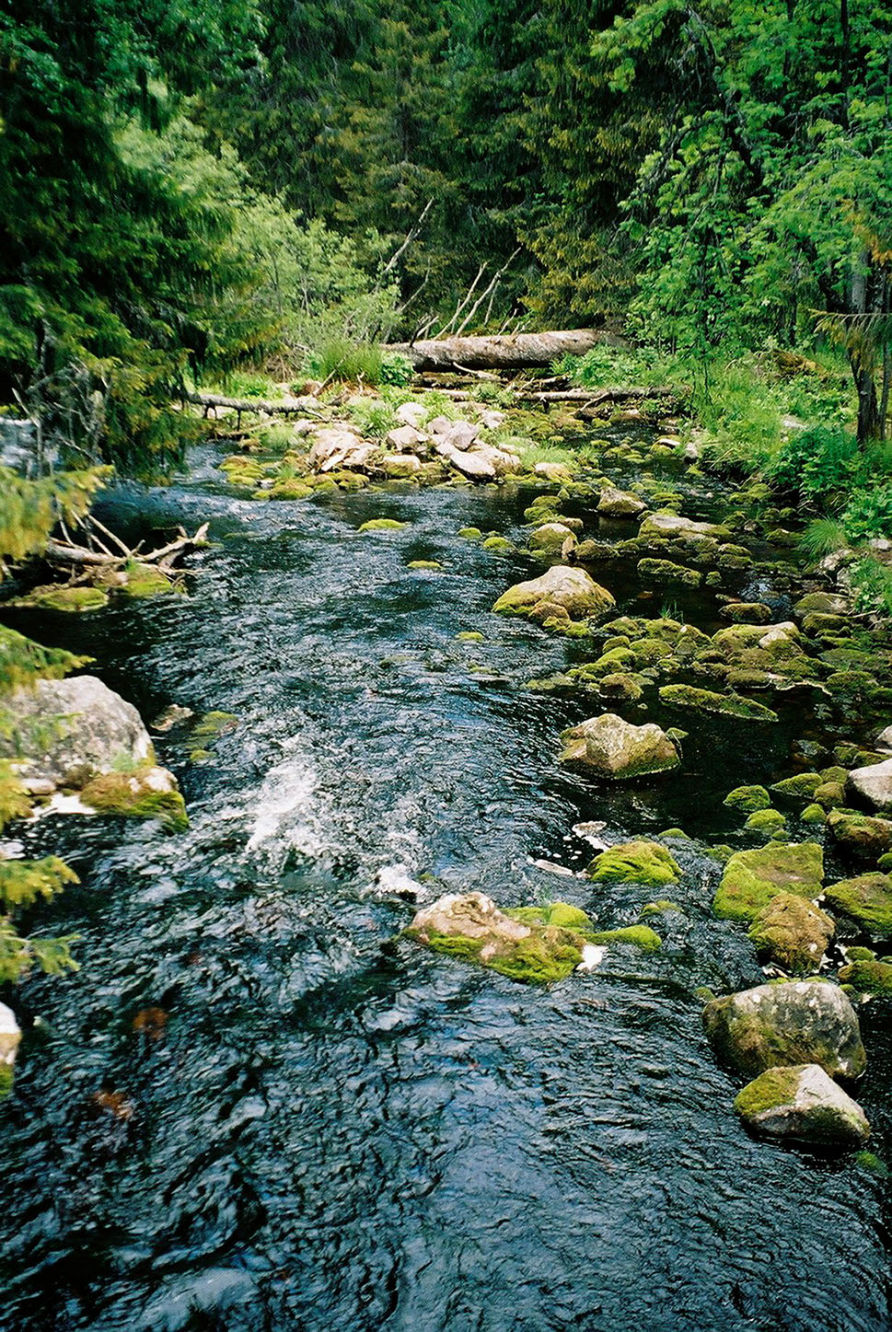
The Stora Njupån stream (catchment area = 23.2 km^2^) discharges into River Fulan 13 km north of Stora Göljån and was not affected by the flashflood. Substrate and riparian vegetation in June 2004 resemble the status at Stora Göljån before the flashflood. Photo: Stefan Löfgren, SLU

In the 3 years following this event, bark- and wood-boring beetles attacked the uprooted trees, while the mortality of standing trees along the riparian forest edge peaked in the second year after the rainstorm (Figs. 3, 5) (Schroeder and Lindelöw 2003). Compared with the flashflood effects on stream geomorphology and possible impacts on the benthic macroinvertebrate fauna in mind, the bark beetle outbreak was of minor importance.

## Materials and methods

### Aneboda IM

#### Tree layer surveys

The forest at Aneboda is monitored every fifth year in permanent circular plots of 314 m^2^ located in a regular grid (50 × 50 m) over the whole catchment. Recordings were scheduled to be performed in 1996, 2001, 2006 and 2011. However, due to the storm in January 2005, and the subsequent massive bark beetle attack observed in 2008, extra inventories were conducted during early spring and late autumn in 2009. The spring inventory gave information about bark beetle effects between 2006 and 2008. The autumn inventory showed bark beetle effects during the growing season in 2009. The inventories are based on individual trees, and five classes were used for classifying the degree of bark beetle infestation (no signs, to dead trees; see Electronic Supplementary Material for details).

#### Soil water, GW, and stream water

During spring, summer, and autumn, SW from Aneboda IM was collected in lysimeters (P80 ceramic cups, 0.3 bar underpressure) in the middle of the B-horizon and at 30–50 cm below the soil surface in organic soils. The lysimeters are located in recharge areas, intermediate zones, and discharge areas (described in Löfgren et al. 2011). Adjacent to the lysimeters, GW was collected in plastic tubes with a diameter of 32 mm, sealed at the bottom and with holes being drilled into the lower 0.5 m in the recharge area (4.2 m), intermediate zone (2.1 m), and discharge area (1.0 m) (Löfgren et al. 2011). Filtered GW samples (<0.45 µm) were collected in winter, spring, summer, and autumn. Stream water was sampled biweekly at the outlet, where daily water discharge was registered. The water was collected in rinsed polyethylene bottles and sent to the laboratory at the Department of Aquatic Sciences and Assessment, SLU for chemical analyses, which were initiated within 1 day after sampling. For further details on methodology and locations of the sampling device, see Löfgren et al. (2011). Annual mass transport of physiochemical compounds was estimated from daily discharge at the stream outlet and linearly interpolated daily concentrations.

The analytic methods are accredited by the Swedish Board for Accreditation and Conformity Assessment (www.swedac.se) and follow Swedish standard methods (see Supplementary material).

### Stora Göljån

#### Benthic macroinvertebrates

Macroinvertebrates were sampled in Stora Göljån in 1992, 1996, and each year between 1997 and 2012. In 1992, 1996, and 2006, only spring sampling occurred, and for 2009, only autumn samples are available. In the other years, macroinvertebrates were sampled both during spring and autumn. After the flashflood, Stora Göljån was plugged approximately 50 m upstream of the original sampling site, and a new stream channel was formed joining Lilla Göljån approximately 250 m downstream of the sampling site. From autumn 1997 and onwards, the benthic macroinvertebrate samples were taken in Stora Göljån approximately 100 m upstream from the original location with similar water discharge and mineral substratum (stones and boulders).

Macroinvertebrates were sampled using a Surber sampler with 0.04 m^2^ sampling surface according to Swedish standard (SS/EN 28 265). In the field, invertebrate samples were preserved in 70 % ethanol. In the laboratory, samples were sorted, and invertebrates identified and counted using dissecting and light microscopes. Organisms were identified to the lowest taxonomic unit possible, generally to species level.

The organic material, also obtained from the Surber sampling, was sorted and determined into different components (algae, detritus, other substrate) and then quantified as ash-free dry weight (AFDW). AFDW (= *M*_105 °C_ − *M*_500 °C_) was quantified as the difference in sample weights after oven drying for 24 h at 105 °C (*M*_105 °C_) and after combustion for 4 h at 500 °C (*M*_500 °C_). Before weighing, the samples were allowed to be cooled in exciccator. Throughout the investigation, the same person (Per Mossberg, Grönbo konsult HB) conducted sampling, sample preparation, identification of taxa, and organic material analyses.

#### Stream water

At Stora Göljån, stream water was sampled on a monthly basis. Before the flashflood, the samples were collected from a bridge just downstream the benthic macroinvertebrate sampling site. After the flashflood, this stream channel was clogged, and the samples were taken from shore just downstream the new benthic fauna sampling site. The water was collected in rinsed polyethylene bottles and before 2009, sent for chemical analysis to the SWEDAC accredited MEANA laboratory in Uppsala. From January 2009 and onwards, the samples were sent to the laboratory at the Department of Aquatic Sciences and Assessment, SLU for chemical analysis. Except for PO_4_^3−^ and TP, the two laboratories used the same analytic methods, and SO_4_^2−^ and Cl^−^ were in fact analyzed at SLU (2014) throughout the monitoring period. At MEANA, PO_4_-P and TP were manually analyzed in a 5-cm quartz cuvette.

### Statistical analyses

The Aneboda IM SW, GW, and stream water N concentration time series were separated into Reference (before January 8, 2005), Storm-felling (January 8, 2005–December 31, 2007) and Bark beetle periods (after January 1, 2008). Differences in N concentrations between periods were tested by the Kruskal–Wallis test followed by a post hoc comparison for each pair using Bonferroni-corrected Wilcoxon test (JMP version 10.0.2d1). Due to the restricted number of observations on TN during the storm-felling period for SW (*n* = 3–15 per season and soil stratum) and GW (*n* = 3 per season and sampling site for all N compounds), we have not tested for seasonality.

In total, eight metrics describing the macroinvertebrate assemblage structure for each sampling occasion (spring and autumn) and year were calculated, and due to statistical differences in macroinvertebrate metrics between seasons, further analysis was separated by season. However, due to the lack of autumn samples and the limited number of spring samples prior to the catastrophic event, we were not able to perform any statistical tests on differences between metrics before and after the storm.

The eight biological metrics were used to analyze possible changes in diversity and composition of macroinvertebrate assemblages. Taxon richness was selected as a qualitative measure of changes in assemblage diversity. In addition, assemblage diversity was calculated as Shannon (Shannon and Weaver 1949) and Simpson (Simpson 1949) diversity as well taxon distinctness. The latter is a diversity measure based on the relatedness of the species within a sample (Warwick and Clarke 2001). Further, a detrended correspondence analysis (DCA) was performed to show temporal changes in taxonomic composition. DCA is an unconstrained ordination technique, which finds the main factors/gradients in large, species-rich, but usually sparse data matrices (ecological community data).

Simple linear regression analyses with metrics versus time were performed to display possible changes in metrics over time, especially focused on the time after the event.

To evaluate the relationships between macroinvertebrate assemblages and environmental factors and to assess which factors that can explain changes in the species assemblage of benthic macroinvertebrates over time, a canonical correspondence analysis (CCA) was performed with physicochemical data, including organic substrate parameters expressed as AFDW (Table 2).

**Table 2:** Minimum, maximum, and mean (±1 SD) values of water chemistry and organic substrate (AFDW mg) variables across all years, but separated in autumn and spring samples at Stora Göljån, used in the CCA

	Autumn				Spring			
	Min	Max	Mean	SD	Min	Max	Mean	SD
pH	5	6.59	6.21	0.47	5.41	6.5	6.12	0.30
Conductivity (mS m^−1^)	0.84	1.92	1.22	0.22	0.84	1.67	1.16	0.22
Alkalinity (meq L^−1^)	−0.02	0.06	0.04	0.02	0	0.06	0.03	0.02
Absorbance (420 nm)	0.03	0.07	0.05	0.01	0.05	0.12	0.08	0.02
TOC (mg L^−1^)	1.5	4.6	2.45	0.80	3.2	5.48	4.3	0.76
NH_4_-N (µg L^−1^)	2	7	4.20	1.52	0	10	3.9	2.38
NO_3_-N (µg L^−1^)	0	197	123.5	47.23	0	156	77.2	38.1
TN (µg L^−1^)	90	245	192.3	40.05	0	357	193.4	73.4
PO_4_-P (µg L^−1^)	1	8	5.76	1.54	0	14	7.02	2.9
TP (µg L^−1^)	5	19	9.61	3.04	0	35	17.8	9.6
Ca (mg L^−1^)	0	1.32	0.91	0.45	0	1.80	0.98	0.5
Mg (mg L^−1^)	0	0.19	0.11	0.05	0	0.18	0.11	0.05
SO_4_ (meq L^−1^)	0.02	0.03	0.03	0.01	0.01	0.04	0.03	0.01
Organic substrate								
Detritus (mg AFDW)	19.8	202.6	52.8	47.3	16.50	180.52	61.9	43.34
*Fontinalis* sp. (mg AFDW)	0	7.74	1.05	2.42	0	96.9	12.3	31.8
*Sphagnum* sp. (mg AFDW)	0	0	0	0	0	26.02	1.72	6.5
Periphyton (mg AFDW)	0	35.4	7.18	9.58	0	35.9	9.04	10.05
Other (mg AFDW)	0	249.3	62.5	83.2	0	431	72.8	114.9

Metrics have been calculated using Asterics version 3.3.1 (http://www.fliessgewaesserbewertung.de); DCA and CCA have been performed using CANOCO 4.5 (ter Braak and Smilauer 1997–2002); and regressions have been made using JMP 10 (SAS 2013).

## Results

### Tree layer effects at Aneboda IM

Before the storm, the fraction of dead Norway spruce trees [standing and downed, >5-cm diameter at breast height (dbh)] in respect of the monitored plots was about 22 % (Fig. 5). The year after the storm, this fraction increased to 32 %. In the following years, the number of dead Norway spruce trees annually increased by ≈5 % mainly as an effect of bark beetles. During the scheduled inventory in 2011, several of the most affected plots were not monitored, as it was considered too dangerous to enter the maze of fallen logs.

The two extra inventories of bark beetle infestation in 2009 showed that bark beetles affected basically all spruces >5 cm dbh. Only 0.7–2 % of the trees were unaffected (total number of investigated trees, *n* = 1053). Half of the trees had minor damages (class 2). Most of these trees did not change class between the two surveys. Between 2006 and 2008, 17 % of the Norway spruce trees died. During the summer 2009, another 8 % of the trees died. In 2011, we recorded that yet another 11 % of the Norway spruce trees had died. However, this percentage should be actually higher as several plots with numerous dead and downed trees were excluded from the 2011 inventory due to safety reasons.

**Fig. 6:**
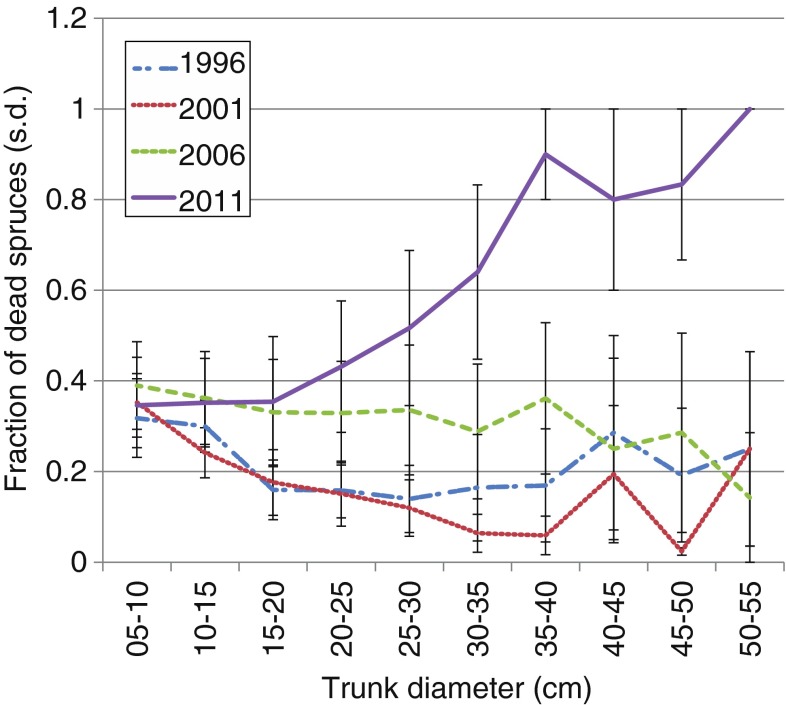
Fraction of dead Norway spruce per trunk diameter class at inventories for different years at Aneboda IM

The highest impact by the bark beetles was on larger trees. For Norway spruce trees with diameters <20 cm, the fraction of dead trees per plot was about the same in all the four inventories. For larger trees, the fraction of dead trees increased drastically in the 2011 inventory (Fig. 6).

### Nitrogen effects at Aneboda IM

**Fig. 7:**
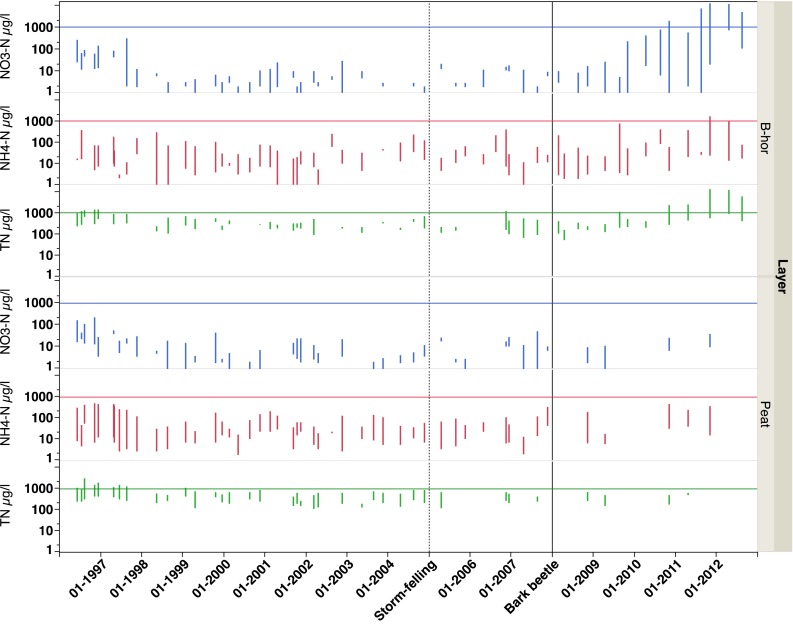
Time series for the period 1996–2012 (*Box-plots*, quantiles) of soil water NO_3_-N, NH_4_-N, and TN concentrations in B-horizon in recharge and intermediate zones (data from eight lysimeters), and in peat in discharge areas (data from four lysimeters) at Aneboda IM. *Horizontal lines* at 1000 µg L^−1^, *vertical dotted line* in January 2005 (storm felling), and *vertical line* in January 2008 (bark beetle attack)

**Fig. 8:**
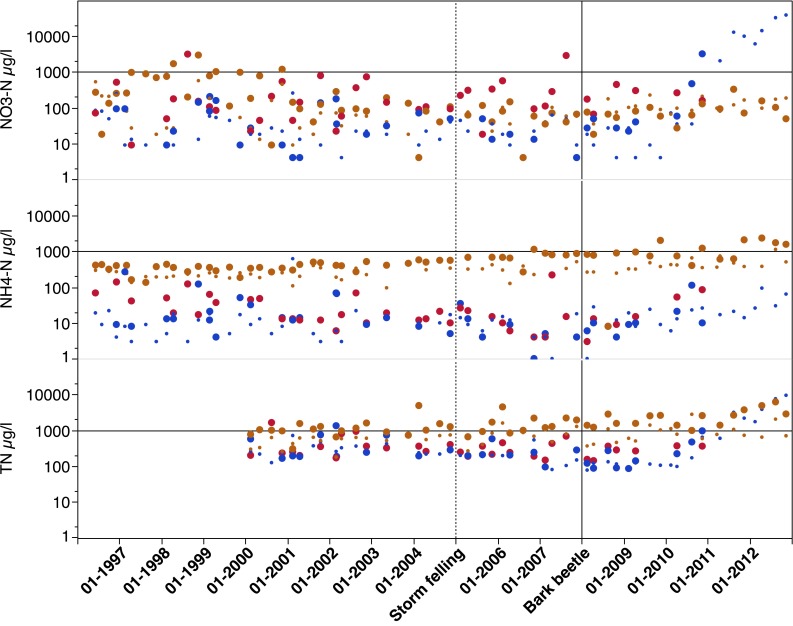
Time series for the period 1996–2012 on GW NO_3_-N, NH_4_-N, and TN concentrations in recharge areas (*red large dots* = 4.2-m soil depth), intermediate zones (*blue* *large dots* = 2.1-m soil depth, *small dots* = 3.2-m soil depth), and discharge areas (*orange large dots* = 1.0-m soil depth, *small dots* = 2.0-m soil depth) at Aneboda IM: *horizontal lines* at 1000 µg L^−1^, *vertical dotted line* in January 2005 (storm felling), and *vertical line* in January 2008 (bark beetle attack)

**Fig. 9:**
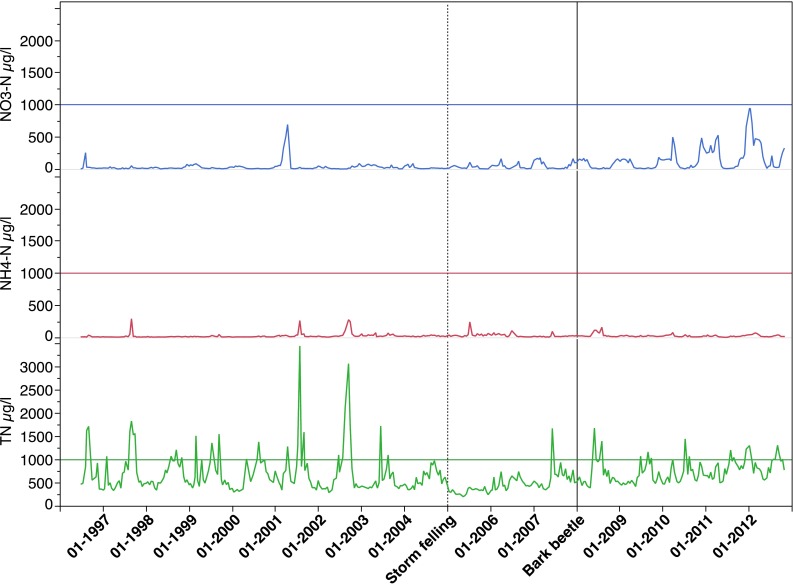
Time series for the period 1996–2012 of the stream water concentrations of NO_3_-N, NH_4_-N, and TN at Aneboda IM: *horizontal lines* at 1000 µg L^−1^, *vertical dotted line* in January 2005 (storm felling), and *vertical line* in January 2008 (bark beetle attack)

There were no indications that the storm felling in January 2005 caused any increased N concentrations in SW in the peaty riparian soils (Fig. 7; Table 3). In the B-horizon, however, the NO_3_-N concentration was significantly higher after the bark beetle attack (*p* < 0.05) (Fig. 7; Table 3). This affected the TN concentrations as well with significantly higher TN concentrations in the order: Reference < Storm felling < Bark beetle period (Table 3). Except for at 3.2-m soil depth in the intermediate zone, showing significantly lower NH_4_-N concentrations during the storm-felling period, no differences between periods were found for N in GW in the recharge areas and intermediate zones. In the discharge area, however, significantly higher NH_4_-N and TN concentrations were found during the bark beetle period both at 1.0-m and 2.0-m soil depths, while excess NO_3_-N concentrations only were found at 1.0-m depth (Fig. 8; Table 3). The period after storm felling also showed significantly higher NH_4_-N, NO_3_-N, and TN concentrations in GW at 1-m soil depth (Table 3). Thus, the higher NH_4_-N and NO_3_-N concentrations in stream water after storm-felling and bark beetle outbreak (Fig. 9; Table 3) were in agreement with relationships between stream water and GW concentrations in the riparian soils as shown earlier (Löfgren et al. 2011). In stream water, the TN concentrations were in the order Storm-felling < Bark beetle < Reference period, implying higher Org-N concentrations before the storm event (Fig. 9).

**Table 3:** Statistical differences (*p* values, Kruskal–Wallis test) between the reference period (before January 8, 2005), the storm-felling period (January 8, 2005–December 31, 2007) and the bark beetle period (January 1, 2008–December 31, 2012) for NH_4_-N, NO_3_-N, and TN in SW, GW, and run off at Aneboda IM: *Rech* recharge area, *Interm* intermediate zone, *Disch* discharge area. Soil depth for GW intake is given in meter; ns denotes nonsignificant *p* values

	NH_4_-N	NO_3_-N	TN
SW B-horizon	ns	<0.05	<0.05
SW Peat	ns	ns	ns
GW Rech 4.2 m	ns	ns	ns
GW Interm 2.1 m	ns	ns	ns
GW Interm 3.2 m	<0.05	ns	ns
GW Disch 1.0 m	<0.001	<0.001	<0.01
GW Disch 2.0 m	<0.001	ns	<0.05
Run off	<0.001	<0.001	<0.001

Except for a few lysimeters in the B-horizon after the bark beetle attack and some GW piezometers throughout the period, the NH_4_-N and NO_3_-N concentrations rarely exceeded 1 mg L^−1^ (Figs. 7, 8). However, after 2010, there were three lysimeters in the B-horizon and one piezometer at 3.2-m soil depth in the intermediate zone showing >5 mg NO_3_-N L^−1^. In the latter, NO_3_-N concentrations up to 9 mg L^−1^ were registered after the bark beetle outbreak. In stream water, the NO_3_-N concentrations rarely exceeded 500 µg L^−1^ and the NH_4_-N concentrations were generally below 100 µg L^−1^ during this period (Fig. 9). The TN concentrations varied between 500 and 1500 µg L^−1^ (Fig. 9). There were no statistically significant differences (*p* > 0.05, Kruskal–Wallis test) between the periods in annual stream export of NH_4_-N, NO_3_-N, and TN at Aneboda IM. The average annual exports were 0.23, 0.06, and 1.93 kg N ha^−1^ year^−1^, respectively (Table 5).

### Macroinvertebrate effects at Stora Göljån

**Fig. 10:**
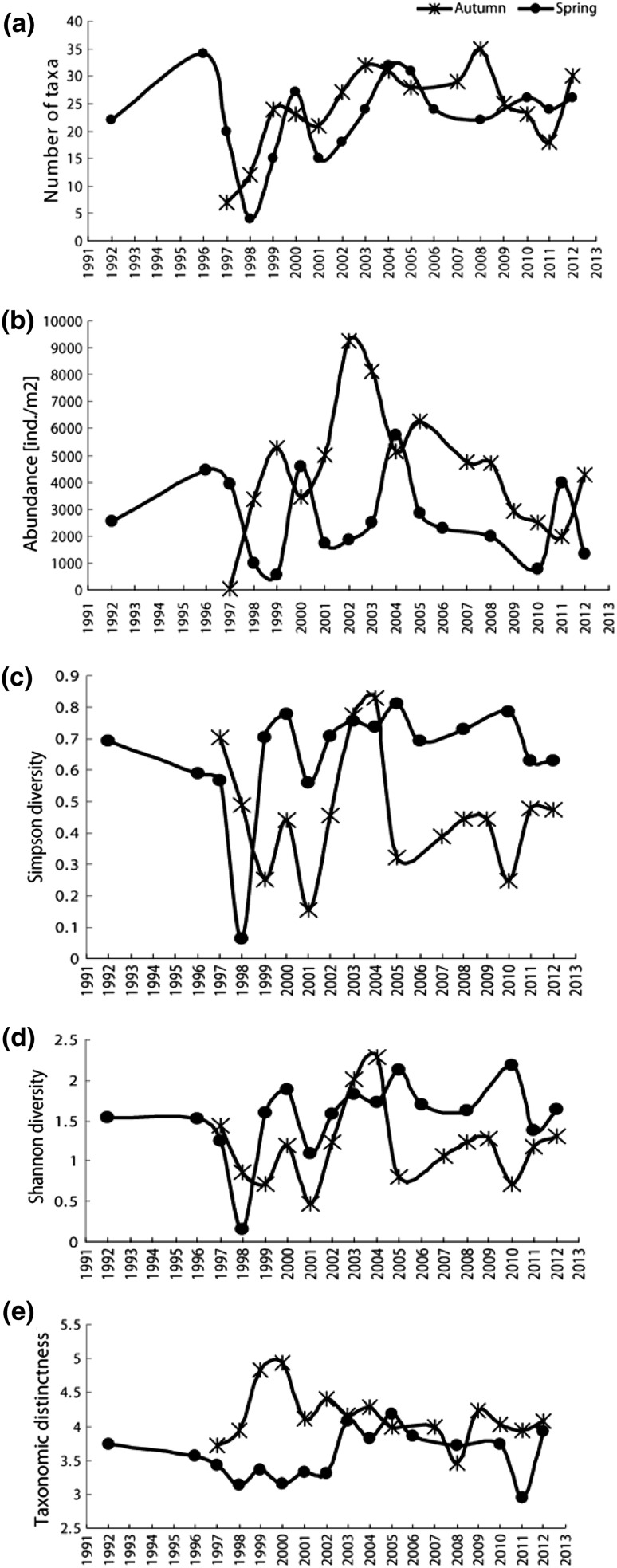
Time series for the period 1996–2012 for benthic fauna assemblage metrics for spring (*circles*) and autumn (*crosses*) in the Stora Göljån stream: **a** number of taxa, **b** abundance (ind. m^−2^), **c** Simpson diversity, **d** Shannon diversity, and **e** taxonomic distinctness

**Fig. 11:**
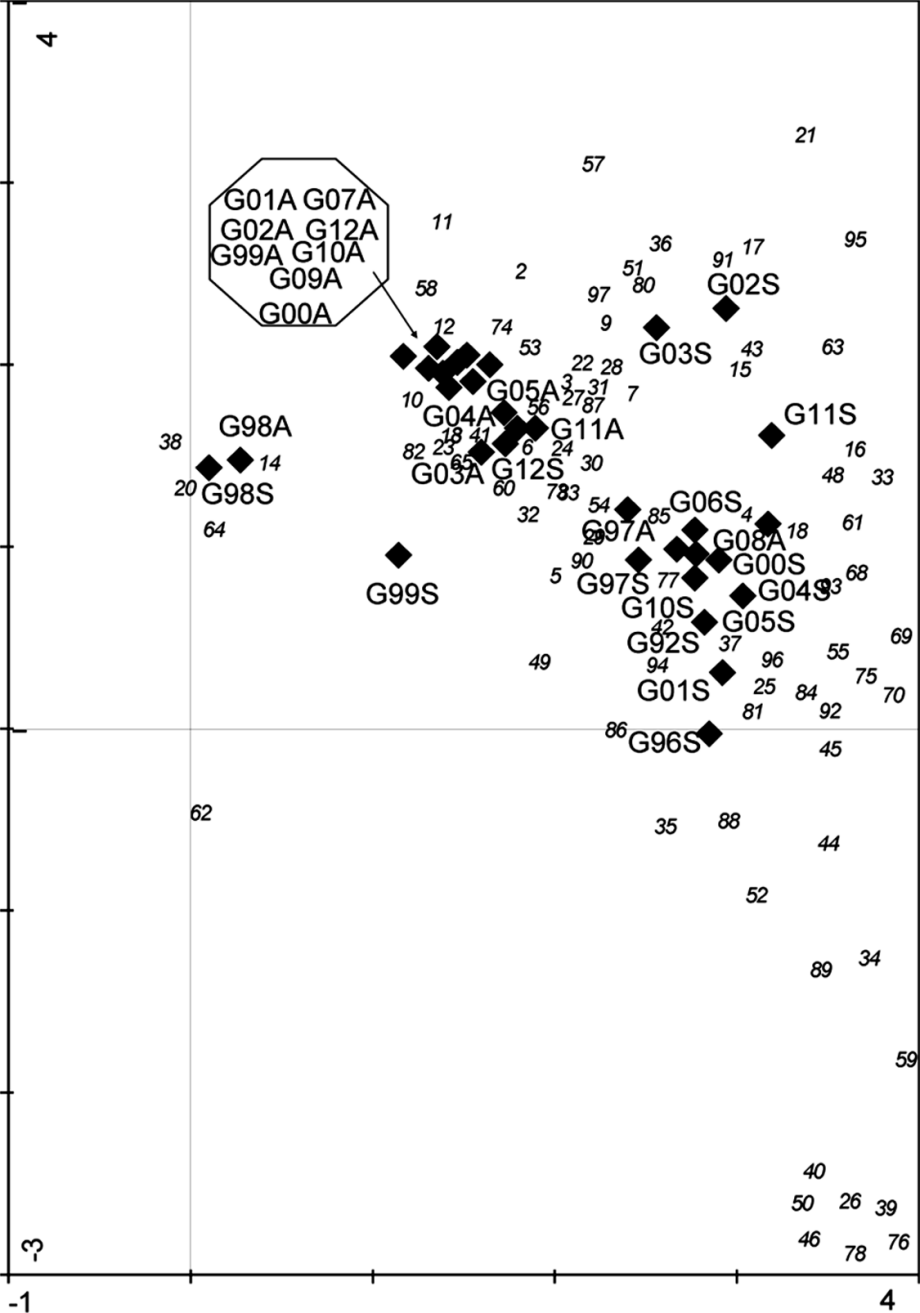
DCA biplot showing samples and species assemblage’s compositions for different years and seasons in the stream of Stora Göljeån. Samples are indicated as G Göljån; sampling year, and season as *A* autumn, *s* spring. Species are represented as *numbers*, see taxa list

The structure and the diversity of the macroinvertebrate assemblage of Stora Göljån were relatively stable and did not vary much through time or between seasons except for the first year after the flashflood (Table 4; Figs. 10a, 11). The number of taxa in autumn ranged from 7 in 1997 (immediately after the flooding) to 35 taxa found in 2008. Number of taxa in spring ranged from 4 taxa found in 1998 (first spring after the event) to 34 taxa recorded in spring 1996. The event in August 1997 clearly caused a severe decrease in species richness in autumn 1997 and spring 1998, but thereafter, species richness increased steadily to pre-event conditions (Fig. 10a).

**Table 4:** All metrics describing the structures of the macroinvertebrate assemblages of Stora Göjlån for the years 1992, 1996, and 1997–2012 for autumn (A) and spring (S) as well as their ranges and mean values. *TD* taxonomic distinctness. The grand means (mean overall years) were significantly different between spring and autumn abundances, Simpson and Shannon diversities, respectively. * *p* < 0.01; ** *p* < 0.001

Year	No. of taxa		Abundance (ind/m^2^)		TD	TD	Simpson diversity		Shannon diversity		Acid index	
	A	S	A	S	A	S	A	S	A	S	A	S
1992		22		2537.5		3.74		0.69		1.54		4
1996		34		4477.5		3.56		0.59		1.52		4
1997	7	20	62.5	3955	3.72	3.43	0.70	0.57	1.44	1.25	3	3
1998	12	4	3360	980	3.93	3.13	0.49	0.06	0.87	0.16	3	1
1999	24	15	5300	565	4.84	3.36	0.25	0.70	0.71	1.59	4	3
2000	23	27	3467.5	4590	4.93	3.15	0.44	0.78	1.20	1.88	4	4
2001	21	15	5030	1742.5	4.11	3.33	0.16	0.56	0.48	1.09	5	3
2002	27	18	9250	1847.5	4.41	3.30	0.46	0.71	1.23	1.58	5	3
2003	32	24	8117.5	2525	4.17	4.07	0.77	0.76	2.01	1.82	4	4
2004	31	32	5150	5777.5	4.28	3.81	0.83	0.74	2.29	1.72	4	5
2005	28	31	6297.5	2842.5	4.00	4.19	0.32	0.81	0.81	2.12	4	5
2006		24		2315		3.86		0.69		1.69		4
2007	29		4782.5		4.00		0.39		1.07		4	
2008	35	22	4730	1985	3.46	3.72	0.44	0.73	1.24	1.63	5	3
2009	25		2932.5		4.24		0.44		1.27		4	
2010	23	26	2490	785	4.03	3.74	0.25	0.79	0.72	2.18	4	4
2011	18	24	1987.5	3975	3.94	2.94	0.48	0.63	1.17	1.38	3	4
2012	30	26	4295	1337.5	4.08	3.93	0.47	0.63	1.31	1.65	4	5
Min	7	4	62.50	565	3.46	2.94	0.16	0.06	0.48	0.16	3	1
Max	35	34	9250	5777.5	4.93	4.19	0.83	0.81	2.29	2.18	5	5
Mean	24.3	22.8	4483.5	2639.8*	4.14	3.58	0.46	0.65**	1.19	1.55*	4	3.69

After the event, the abundance of macroinvertebrates showed high interannual variability, with some peaks like in autumn 2002, when the highest abundance (9000 individuals m^−2^) was found (Fig. 10b). In contrast to the number of taxa, grand mean abundances were different between seasons (4484 in autumn vs. 2639 individuals m^−2^ in spring, *p* = 0.02) (Table 4). In autumn 1997, directly after the flooding, the lowest abundance (62.5 individuals m^−2^) was recorded. Spring abundance was generally lower, and records ranged from 565 individuals m^−2^ in 1999 (i.e., 1.5 years after the event) to over 5700 individuals m^−2^ found in 2004. However, after 2004, both spring and autumn abundances showed a slight decreasing trend reaching somewhat lower numbers at the beginning of the actual decade.

Both Simpson and Shannon diversities also showed great interannual variability with the spring diversity being somewhat, but not significantly, higher than the diversity at autumn (Fig. 10c, d). The grand mean diversity values were different between seasons for both Simpson (0.46 vs. 0.65; *p* = 0.006) and Shannon diversities (1.19 vs. 1.55, *p* = 0.03), but not for taxonomic distinctness (4.14 vs. 3.58) (Table 4). In autumn, minimum Simpson and Shannon diversities (0.16 and 0.48, respectively) were recorded in 2001, while maximum Simpson and Shannon diversities (0.83 and 2.29, respectively) were recorded in 2004. In spring 1998, a few months after the event, the lowest Simpson (0.06) and Shannon (0.16) diversities were recorded. Both indices peaked many years later (2005 and 2010, respectively). Maximum Simpson diversity coincided with the highest taxonomic distinctness obtained in spring 2005, i.e., 4.19 (Fig. 10e). Taxonomic distinctness was slightly higher in autumn, but not significantly different from the spring. Autumn taxonomic distinctness values ranged from 3.46 (2008) to 4.93 (2000) and from 2.94 (2011) to 4.19 (2005) in spring, but the slightly higher distinctness in autumn was not significantly different from that in spring. DCA of individual years and seasons showed distinct grouping in macroinvertebrate assemblage’s composition in Stora Göljån (Fig. 11).

The first DCA axis explained 28 % (eigenvalue = 0.72) of the total inertia, i.e., the total variance explained (eigenvalue = 2.56) in assemblage’s composition and clearly separating seasons (*t* test of axis 1 DCA scores, *p* < 0.001). The second axis accounted for another 9.7 % of the variability, and was more related to within-season interannual variability. Basically, there are two groups showing distinct assemblage’s compositions for autumn and spring, respectively and some samples separated from these groups having deviating macroinvertebrate assemblage’s structures (G99S, G02S, G03S, G11S, G98A, and G98S, Fig. 11). However, there was also an overlap, where the invertebrate composition in autumn (G97A and G08A) was more similar to the spring macroinvertebrate composition and vice versa, while the composition in spring 2012 (G12S) was more similar to that of the samples obtained in autumn.

**Fig. 12:**
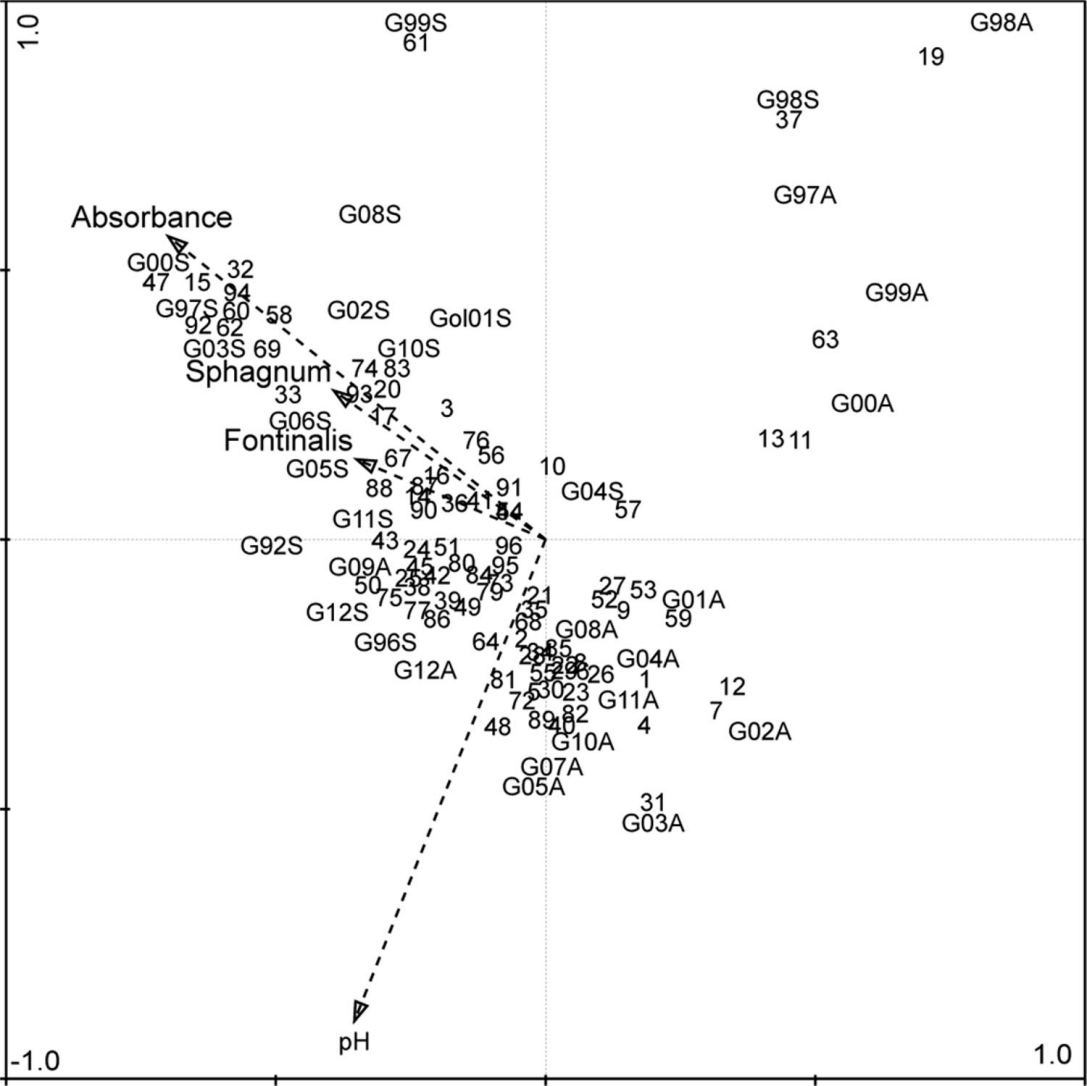
CCA triplot showing samples and species assemblage’s compositions for different years and seasons in the stream of Stora Göljån as well as the four explanatory factors significantly explaining assemblage structure variability. Samples are indicated as G Göljån; sampling year, and season as *A* autumn, *S* spring. Species are represented as *numbers*, see taxa list

CCA with water chemistry and organic substrate as explanatory variables and macroinvertebrate assemblages (species compositions) of spring and autumn samples as independent variables showed that mosses, water color (absorbance at 420 nm), and pH were the most important determinants of assemblage structure (Fig. 12). The first two CCA axes explained 55.8 % of the variation in invertebrate composition (axis 1 explained 28.8 %, and the second axis accounted for another 27 % of the variability). As already shown in the DCA of Fig. 11, spring and autumn samples were clearly separated, where pH was associated with autumn samples, and water color was associated with spring samples. The absence of *Fontinalis* sp. and *Sphagnum* sp. in spring, especially in the years 1997, 2000, 2003, 2005, and 2006, was positively correlated to spring assemblage structure.

## Discussion

### Evaluating stochastic climatic events: A methodological challenge

Evaluating the effects of stochastic weather events is a challenge. Before an event, you do not know where or when it occurs. Hence, it is impossible to use scientific methods based on reference periods and “control” and “treatment” sites for such studies. By chance, two Swedish monitoring sites were hit by extreme weather events, contributing with pre- and post-event data. The assessments are restricted to the types of data collected, and the Stora Göljån macroinvertebrate study suffers from poor descriptions of streambed characteristics at the two sampling sites. However, an earlier study on geomorphologic effects in Stora Göljån (Borgström et al. 1999) showed that similar weather events have occurred historically and that the sediments in the lower parts consist of the locally produced and the recently deposited stones and boulders of varying but similar sizes (Figs. 2, 3). Hence, from a minerogenic and texture point of view, the pre- and post-event sampling sites are rather similar. Besides the enormous translocation of minerogenic matter, the largest difference in Stora Göljån was the vegetation cover (cf. Fig. 5), which was completely removed by the intense erosion during the flashflood. Accumulation of the newly formed large woody debris (LWD) along the shores (Fig. 3) was another difference. LWD was also present before the event, but in much lower quantities and probably of another substrate quality compared with the post-event conditions (cf. Fig. 5). Hence, the shift in macroinvertebrate sampling site between pre- and post-event periods must have had marginal impact on the results compared with the flashflood-created substrate changes.

### Nitrogen effects at Aneboda IM

The storm Gudrun had a relatively low impact on the tree layer at Aneboda IM. The storm-felling event increased the fraction of standing and downed (>5 cm dbh) Norway spruce trees from 22 to 32 %, while the following bark beetle infestation had a much larger effect with an annual 5 % increase of dead trees. Especially larger Norway spruce trees (>30 cm dbh) were killed, and 65–100 % of them were dead in 2011 (Fig. 6). The destruction of the mature spruce canopy together with the large disturbances of the soil by uprooted trees theoretically favors reduced evapotranspiration, increased mineralization of organic matter, and thereby increased losses of N to surface waters. However, these changes in combination with changes in light regime will most likely have stimulating effects on the ground- and field-layer vegetations having the potential to compensate for the excess N availability in the soil system (Mikkelson et al. 2013). Unpublished results from the inventories in summer 2013 confirm that the drastic perturbation of the abiotic environment have resulted in abrupt changes in the field layer. For example, the wavy hair-grass (*Deschampsia flexuosa* L.) has changed from a mean cover of about 1 % before 2010 to >50 % in several monitoring plots. Another major change is the appearance of raspberry (*Rubus idaeus* L.) in high densities. Both these species are nitrophilic and commonly associated with clear-cuts. At Aneboda IM, the large increase in density of these species is probably an effect of the change in the light regime from a relatively dark understory to fully exposed ground (Fig. 4) and good availability of inorganic N in the soils.

In this study, increased concentrations of NO_3_-N were only found in SW in the B-horizon and in shallow GW (1 m) in the discharge areas. The riparian soils also exhibited excess NH_4_-N concentrations related to the death of Norway spruce trees (Table 3). The leakage of inorganic nitrogen to the stream, however, was relatively moderate, and the NH_4_-N and NO_3_-N concentrations rarely exceeded 100 and 1000 µg N L^−1^, respectively. This increase was to a large extent balanced by lower Org-N concentrations (Fig. 9). In fact, it was not possible to observe any statistically significant effects on the stream N export coupled to the storm-felling or bark beetle outbreak periods (Table 5). These relatively few and modest effects on the N dynamics are in agreement with other studies performed in less N-enriched environments in North America, but clearly deviate from the results found in “N-saturated” systems in Central Europe (see Mikkelson et al. 2013 and references therein).

**Table 5:** Annual transports (kg ha^−1^ year^−1^) of nitrate (NO_3_-N), ammonium (NH_4_-N), and total nitrogen (TN) during the period 1997–2012 at Aneboda IM. The years, with storm felling 2005 and intense bark-beetle infestation (2008), are highlighted by italics

	NO_3_-N	NH_4_-N	TN
1997	0.03	0.02	1.23
1998	0.07	0.04	2.76
1999	0.13	0.04	1.97
2000	0.08	0.03	2.00
2001	0.39	0.06	1.79
2002	0.08	0.07	1.98
2003	0.07	0.05	0.95
2004	0.07	0.08	2.23
*2005*	*0.06*	*0.09*	*0.73*
2006	0.13	0.08	1.62
2007	0.32	0.07	2.23
*2008*	*0.32*	*0.10*	*2.11*
2009	0.19	0.05	1.77
2010	0.64	0.07	2.69
2011	0.80	0.06	2.82
2012	1.11	0.09	2.73
Mean	0.23	0.06	1.93

However, some of our 5-year long time series from SW and GW after the bark beetle infestation indicate that the net-loss of NH_4_-N and NO_3_-N may not have reached a maximum in certain areas of the catchment (Figs. 7, 8) and that the prolonged leakage may be expected to the stream showing the highest NO_3_-N concentrations in the autumn of 2011 (Fig. 9). Clow et al. (2011) did not find increased NO_3_-N concentrations in Colorado streams within 9 years from mountain pine beetle-induced tree mortality, but the TN concentrations increased. Vrba et al. (2014) documented excess NO_3_-N concentrations during the 6 years in lakes in the German part of Bohemia, and Oulehle et al. (2013) found similar results in lakes on the Czech side. Both the latter studies represent N enriched environments. While the N deposition in throughfall in Bohemia (14 kg N ha^−1^ year^−1^) exceeded N in bulk deposition by 30–40 % (Oulehle et al. 2013), the N in throughfall (2.4 kg N ha^−1^ year^−1^) was significantly lower compared with bulk deposition (7.5 kg N ha^−1^ year^−1^) at Aneboda IM (Table 1). This strongly indicates N-limited forest ecosystems at Aneboda IM.

Besides a major change in the field vegetation (unpublished data) and modest atmospheric N deposition, the remaining living trees (cf. Fig. 4) may have a profound impact on the N dynamics, explaining the N-concentration heterogeneity in SW and GW. Another factor not to overlook is the tremendous amount of LWD that has been accumulated within the catchment. It is well known that LWD with high C/N ratios may act as N sinks during decomposition (Hyvönen et al. 2000; Brais et al. 2006; Palviainen et al. 2010). Especially the stumps seem to accumulate N, and during the first 5 years after harvesting, a study from southern Finland showed about twofold increases in stump N content, corresponding to 2.2–2.8 and 1.4–1.8 kg N ha^−1^ year^−1^ in Scots pine and Norway spruce stands, respectively (Palviainen et al. 2010). Adding boles and large branches, the N uptake by LWD may constitute a significant factor for explaining why Aneboda IM still exhibits a fairly closed N budget with limited excess leakage of N to the stream.

We have to reject our first prediction of no impact by storm felling and bark beetle outbreak on the N concentrations in the Aneboda IM stream. The SW and GW data indicate that excess NO_3_-N leakage to the stream may continue for still some more years to come. However, the NO_3_-N concentrations will probably maintain at relatively low levels not tangibly increasing the export of N from the catchment compared to those levels expected to find if it was covered with a vital Norway spruce forest (Table 5).

### Macroinvertebrate effects at Stora Göljån

The extreme rain event at Mount Fulufjäll in 1997 generated a tremendous flashflood in Stora Göljån, which severely altered the streambed geomorphology, uprooted trees, and other vegetation along broad riparian strips. Total macroinvertebrate density and taxa richness decreased dramatically shortly after the event in autumn 1997 and remained low also at the first post-event investigation in spring 1998 (Table 4). However, benthic fauna recovered quite rapidly reaching almost pre-event densities, richness, and diversity within the first post-event year (Table 4). This quick recovery to pre-event numbers of taxa and also diversity was somewhat unexpected at a first glance. However, flow peaks annually affect the Stora Göljån stream during snowmelt, although their intensities are not at all comparable with the enormous forces created by the 1997 flashflood. This annually recurring disturbance pattern may have contributed to the adaptations that the macroinvertebrate assemblage has evolved to show resilience to discharge events (e.g., Lytle and Poff 2004).

Further, the assemblage structure did not change after the flashflood. This is also unexpected because of the severe damage to the riparian zone vegetation and the substrate. One would expect a change of the proportion of certain functional feeding groups within the assemblage. For instance, due to the reduced or loss of shading caused by the damage to the riparian forest, the proportion of grazers is expected to increase due to the expected increase of periphytic biomass, whereas shredders should decrease due to the reduced input of allochthonous organic material. However, in our study, neither the algal biomass increased nor the proportion of either grazers or shredders changed significantly after the flashflood. This finding is in contrast to other studies, where flashfloods altered the streambed and also the riparian zone in similar ways as at Stora Göljån, but where stream macroinvertebrate community composition changed tremendously, and the recovery of taxon richness and density was highly delayed (e.g., Minshall et al. 1997; Vieira et al. 2004). However, the recovery might be rapid even after large, single floods due to traits allowing for fast recolonization. For instance, no significant difference has been found between pre- and post-flood benthic assemblages within a 2-month-period of a single flood within a 100-year period in River Isar, Germany (Hering et al. 2004).

In our study, the recovery occurred within the first post-event year. The quick recovery might be explained not just by a high resilience of the assemblage, but more because a new stream channel was formed by the flashflood offering new habitats with resources relatively quickly available and thus facilitating recolonization from water sources nearby. Indeed, sampling post-flood was performed in this new stream channel, not far away from the confluence of the Stora Göljån stream with river Fulan, from which colonists, especially insects, may disperse. The river may act as an excellent source, and the more or less open land due to the cutting of a large amount of forest within the Fulan catchment may have contributed to easier and quicker dispersal of flying insect species to the new habitats. Drifting from upstream not so seriously damaged tributaries on the plateau may also have acted as refuges, facilitating the recolonization.

Hence, we have to reject also our second prediction of strong and long-term impact on benthic macroinvertebrates in Stora Göljån due to the geophysical disturbances by the flashflood. However, more subtle impacts may have had longer duration, but this cannot be evaluated due to pre-event data constraints.

## Conclusions

Long-term monitoring data from Aneboda IM and Stora Göljån make it possible to assess the effects of two extreme weather events. The results indicate long-term (>5 years) increased NH_4_-N and NO_3_-N concentrations in aqueous media at Aneboda IM primarily induced by the bark beetle infestation following the storm. The enhanced N concentrations were, however, relatively modest (generally <1 mg L^−1^) probably as a result of low N deposition, a remaining cover of N-limited young trees, and compensatory factors such as rapidly evolving field vegetation and N retention by LWD with a high C/N ratio. The extreme flashflood at Stora Göljån, completely changing the morphology of the stream channel, induced only short-term (1 year) effects on the benthic fauna diversity and composition. The quick biological recovery may be explained by the creation of new habitats with resources relatively quickly available and recolonization from water sources nearby.

Both these case studies illustrate that Swedish freshwater systems can have a remarkable resilience and fast recovery, even after massive disturbances. The results are partly in contrast to extreme weather event studies elsewhere showing much more dramatic effects on water chemistry and benthic invertebrates.

## Electronic supplementary material

Below is the link to the electronic supplementary material.

Supplementary material 1 (PDF 55 kb)
