# Supplementary material for: Long-term effects on nitrogen and benthic fauna of extreme weather events: Examples from two Swedish headwater streams
Source: Ambio. 2014 Nov 15;43(Suppl 1):58–76. doi: 10.1007/s13280-014-0562-3 (PMC4235926; doi:10.1007/s13280-014-0562-3)
Supplement: Supplementary file 1 — Supplementary material 1 (PDF 55 kb) [file 13280_2014_562_MOESM1_ESM.pdf]

AMBIO

ELECTRONIC SUPPLEMENTARY MATERIAL

This supplementary material has not been peer reviewed.

Long-term effects on nitrogen and benthic fauna of extreme climatic events: Examples from two Swedish headwater streams

Stefan Löfgren, Ulf Grandin, Sonja Stendera

Classification system for the degree of bark beetle infestation

During the spring inventory in 2009, the degree of bark beetle infestation was quantified using the following scale:

1 = No visible signs of infestation, all needles green.

2 = Traces of bark beetles, most needles green and all bark intact.

3 = Tree alive, but with clear signs of bark beetle infestation. Significant amount of brown needles or bark partly flaked.

4 = Dead 2008. Only brown needles mainly still attached to twigs and branches.

5 = Dead before 2008. No needles left on the tree.

For the autumn inventory the last two classes were changed to

4 = Dead 2009. Only brown needles mainly still attached to twigs and branches.

5 = Dead before 2009. No needles left on the tree.

Water chemical analyses

pH was measured in a through-flow cuvette using a Radiometer PHM 210 Precision pH-meter at ambient pCO_2 pressure (pH). Total organic carbon (TOC) was measured using a Shimatzu TOC 5050 analyzer with ASI-502 sample injector following acidification. Analysis of calcium (Ca^{2+}), magnesium (Mg^{2+}), sodium (Na^+) and potassium (K^+) was performed by ICP-OES (Varian Vista Ax Pro), while sulfate (SO_4^{2-}) and chloride (Cl^-) were analyzed by ion chromatography (LDC ConductoMonitor III). Ammonium (NH_4^+, indofenol method), nitrate (NO_3^-, sulphanil amid method after Cd-reduction), total phosphorus (TP, molybdenium method after persulfate digestion) and phosphate (PO_4^{3-}, molybdenium method) were photometrically analyzed (Bran Luebbe Autoanalyzer 3). Before 2005, total nitrogen (TN) was calculated from the sum of Kjeldahl-N, nitrite and nitrate. Between 2005-2006, TN was

analyzed after persulfate digestion (Technicon Traacs 800) and from 2007 and onwards measured using a TNMI-module equipped Shimatzu TOC-VCPH analyzer. Further information on the analytical methods, including analytical precision and limits of detection, can be found at the website of the Department of Aquatic Sciences and Assessment (SLU 2014).
